# Association of levels of interleukin 17 and T-helper 17 count with symptom severity and etiology of chronic heart failure: a case-control study

**DOI:** 10.3325/cmj.2018.59.139

**Published:** 2018-08

**Authors:** Zahra Rahmati, Ali Akbar Amirzargar, Samaneh Saadati, Farzaneh Rahmani, Mohammad Jafar Mahmoudi, Zahra Rahnemoon, Vajiheh Eskandari, Fatemeh Gorzin, Mona Hedayat, Nima Rezaei

**Affiliations:** 1 Department of Immunology, School of Medicine, Tehran University of Medical Sciences, Tehran, Iran; 2 Molecular Immunology Research Center, Tehran University of Medical Sciences, Tehran, Iran; 3 Research Center for Immunodeficiencies, Pediatrics Center of Excellence, Children's Medical Center, Tehran University of Medical Sciences, Tehran, Iran; 4 NeuroImaging Network (NIN), Universal Scientific Education and Research Network (USERN), Tehran, Iran; 5 Department of Cardiology, Amir Alam Hospital, Tehran University of Medical Sciences, Tehran, Iran; 6 Cardiac Heart Center, School of Medicine, Tehran University of Medical Sciences, Tehran, Iran; 7 Division of Immunology, Boston Children's Hospital, Harvard Medical School, Boston, MA, USA; 8 Network of Immunity in Infection, Malignancy and Autoimmunity (NIIMA), Universal Scientific Education and Research Network (USERN), Boston, MA, USA; 9 Network of Immunity in Infection, Malignancy and Autoimmunity (NIIMA), Universal Scientific Education and Research Network (USERN), Tehran, Iran

## Abstract

**Aim:**

To assess the association between the levels of interleukin 17 (IL-17) and T-helper 17 count and symptom severity and etiology of chronic heart failure.

**Methods:**

This single-center prospective case-control study, conducted from December 1, 2015 to January 1, 2017 in Tehran Heart Center, evaluated gene expression of IL-17, relative count of (CD4^+^IL17^+^) Th17 cells and CD4^+^ helper T-cells in peripheral blood mononuclear cells of 42 patients with CHF and 42 matched controls. A multiple regression model assessed the predictors of peripheral IL-17 expression and Th17 count in patients with CHF.

**Results:**

IL-17 expression was increased in patients with CHF, both at baseline and after stimulation. IL-17 and Th17 counts were higher in patients with advanced New York Heart Association (NYHA) functional class (class IV) than in controls and patients with class I. Th17 cell population expanded in patients with CHF, more prominently in patients with class IV than in controls and patients with class I, regardless of the ischemic or non-ischemic CHF origin. Multiple regression model showed that NYHA was the only meaningful predictor of IL-17 levels and Th17 count.

**Conclusion:**

We demonstrated the lymphocytic origin of IL-17 production in advanced CHF and the ability of disease severity to predict IL-17 levels.

Oxford Centre for Evidence-based Medicine level of evidence: 3.

The hallmark feature of congestive/chronic heart failure (CHF) is progressive myocardial fibrosis and cardiac remodeling (1). After myocardial ischemic injury, cardiomyocytes and cardiac fibroblasts secrete large amounts of transforming growth factor beta (TGF-β) and interleukin-10 (IL-10) to ensure proper wound healing and fibrosis (2). A simultaneous increase in peripheral expression of TGF-β warrants the polarization of naďve T-cells into regulatory phenotype and infiltration of profibrotic monocytes/macrophages into the injured site, precipitating cardiac remodeling in an extrinsic manner (3). This happens while simultaneous up-regulation of proinflammatory cytokines, interleukin-1β (IL-1β), tumor necrosis factor alpha (TNF-α), and interleukin 6 (IL-6) within the myocardium and in peripheral blood mononuclear cells (PBMC) counteracts the immunoregulatory phenotype of regulatory T-cells (Treg). This proinflammatory milieu acts in favor of maturation and expansion of T helper 17 (Th17) cells in advanced stage of CHF (4).

Along with disease progression and progressive expression of proinflammatory cytokines, loss of forkhead box P3 (FOXP3) expression gives rise to maturation of regulatory T-cells without immunosuppressive phenotype that retain their profibrotic features. According to Bansal et al (5), the increased Th17/Treg ratio sustains the proinflammatory milieu and progressive cardiac remodeling. In line with this, repletion of interleukin 17 (IL-17A) in IL-17A knockout mice aggravated acute and chronic cardiac remodeling, increased infarct size, and induced cardiomyocytes apoptosis in a myocardial infarction model (1). Feng et al (6) reported successful inhibition of cardiac remodeling in terms of collagen type I and III accumulation in a model of isoproterenol-induced chronic heart failure, through intravenous administration of anti-IL-17 antibodies. This effect was mediated through the suppression of matrixmetaloproteinase 1 (MMP-1) expression.

Li et al (7) demonstrated that increased IL-17 expression in CHF patients with advanced class (class III and IV) of New York Heart Association (NYHA) staging closely correlated with levels of pro-brain type natriuretic peptide, a known prognostic risk factor in these patients. Th17 are the most abundant source for IL1-7 production. We investigated the role of IL-17 levels and relative Th17 counts in peripheral blood mononuclear cells (PBMCs) of patients with different stages of CHF. We hypothesized that IL-17 expression was elevated in patients with CHF, and this increase was due to expansion of Th17 cells over other T helper populations. Also, we hypothesized that the possible IL-17 up-regulation or Th17 expansion correlate with disease stage and duration of CHF.

## Materials and methods

### Study design and participants

This was a single-center prospective case-control study conducted from December 1, 2015 to January 1, 2017 in Tehran Heart Center, a national cardiology referral center.

Adopting a statistical power of 80% (Z_β_ = 0.84) and significance threshold of 95% (Z_α/2_ = 1.96), we used standard deviations (SD) and mean differences from a similar study (8) for baseline and stimulated IL-17 levels, respectively (8), and the methods described elsewhere (9). This yielded a number of 45 participants per group (case:control ratio was 1:1). *Post-hoc* power analysis for flow cytometry and polymerase chain reaction (PCR) analyses were 95% and 89%, respectively.

We devised a case-control design, enrolling 42 patients with CHF (24 men; mean age: 55.3 ± 5.76 years) from referrals to the Tehran Heart Center as the case group. A total of 42 age- and sex-matched healthy individuals (26 men; mean age: 54.35 ± 0.74 years) were included from Iranian Blood Transfusion Bank during the same period as the control group (Table 1).

**Table 1:** Baseline characteristics of patients with chronic heart failure (CHF) and controls*

	Patients with CHF (n = 42)				Controls (n = 42)	*P*
**Age** (years; mean ± standard deviation)	55.3 ± 5.76				54.35 ± 0.74	NS
**Sex (male:female)**	24:18				26:16	NS
**Mean systolic blood pressure (mmHg;** mean ± standard deviation**)**	143 ± 30 (38 with systolic hypertension)				135 ± 28	<0.01
**Mean diastolic blood pressure (mmHg;** mean ± standard deviation**)**	103 ± 20 (38 with diastolic hypertension)				83 ± 21	<0.01
**Serum N-terminal pro brain natriuretic peptide (pg/mL;** mean ± standard deviation**)**	201.0 ± 67.3				-	-
**Disease duration (years;** mean ± standard deviation**)**	14.05 ± 8				-	-
**Mean LVEF (%;** mean ± standard deviation**)**	24.6 ± 1.13				-	-
**NYHA functional class**	I	II	III	IV	-	-
**Ischemic**	3	8	6	13		
**Non-Ischemic**	3	3	6	0	-	-
**Severe valvular disease**	MS: 2	AS: 3	TS:0	PS: 0	-	-
	MR: 0	AR: 1	TR:0	PR: 0		

*LVEF – left ventricular ejection fraction; MS – mitral valve stenosis; MR – mitral valve regurgitation; AS – aortic valve stenosis; AR – aortic valve regurgitation; TS – tricuspid valve stenosis; TR – tricuspid valve regurgitation; PS – pulmonary valve stenosis; PR – pulmonary valve regurgitation.

The diagnosis of CHF in the case group was made on clinical grounds and the patient’s most recent echocardiography (performed within a maximum of 6 months), and if not available echocardiography performed at the time of sampling, in which the left ventricular ejection fraction (LVEF) had been less than 45% and signs of structural heart disease, including but not limited to left ventricular eccentric hypertrophy, left ventricular remodeling, dilation, hypokinesia, etc. were evident (10). Patients with clinical symptoms, LVEF between 45%-50%, and the mentioned echocardiographic signs were also included. We excluded patients with LVEF≥50%, ie, heart failure with preserved ejection fraction. Therefore, in our study CHF refers to left ventricular systolic dysfunction alone, or systolic in combination with diastolic left ventricular dysfunction. Mean LVEF of the patients was 24.6 ± 1.13% and mean systolic/diastolic blood pressure was 143 ± 30 mm Hg/103 ± 20 mm Hg. Each patient was examined by two specialists to confirm disease status.

**Figure 1:**
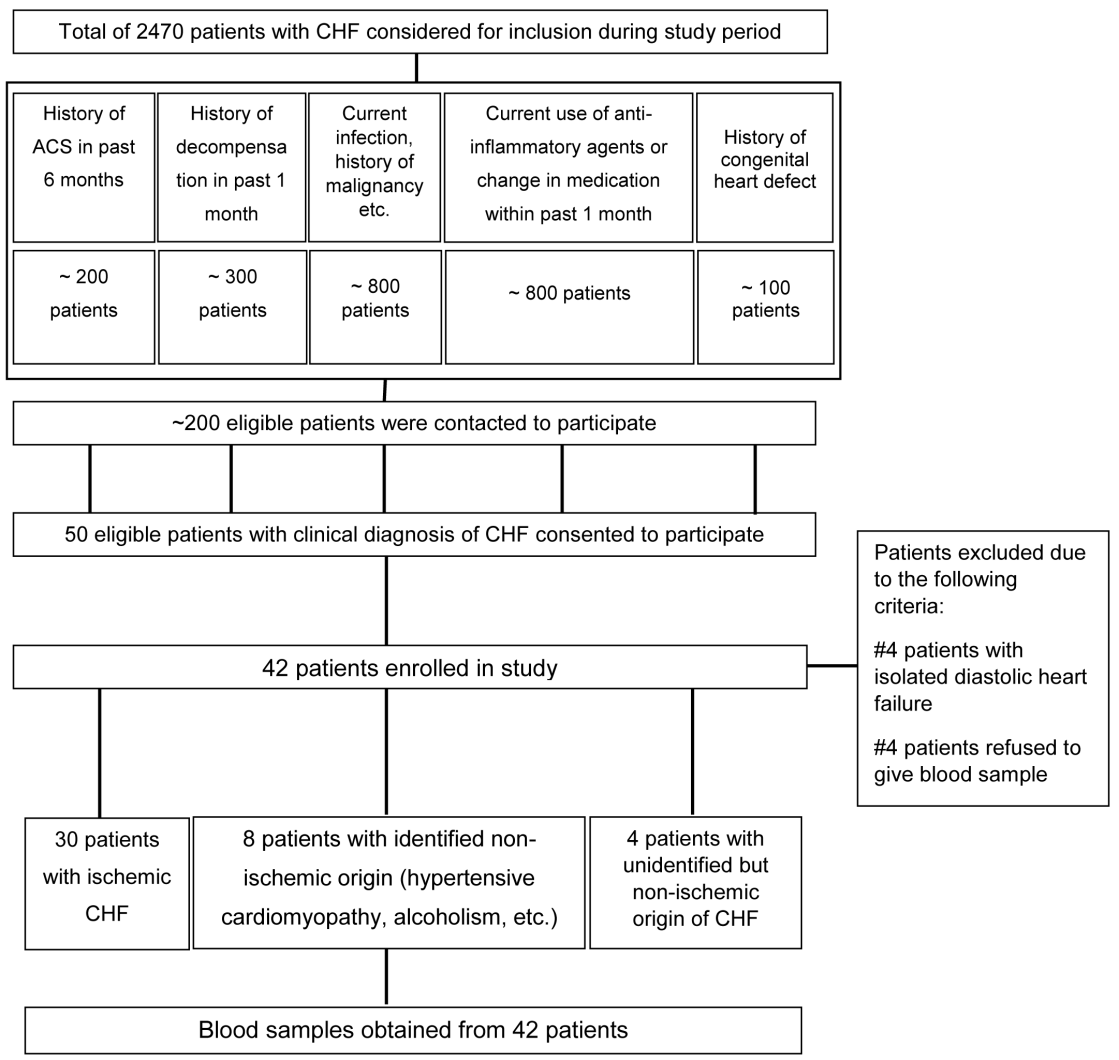
Selection flowchart for patients with congestive heart failure. ACS – acute coronary syndrome; CHF – chronic heart failure.

Patients were excluded if they were in decompensated heart failure phase at the time or within the last 1 month of sampling, or if they had experienced acute coronary syndrome within the last 6 months. Additional exclusion criteria were: 1) current or recent (within the last 1 month) history of infections or presence of fever, 2) co-morbid malignancies, 3) use of non-steroidal anti-inflammatory agents, corticosteroids, or biological drugs at the time of sampling, 4) a recent (within last 1 month) change in medication regimen, and 5) history of chemotherapy with cardiotoxic agents or clear history of exposure to cardiotoxic/profibrotic chemicals (eg, anthracyclines, alkylating agents, etc), or 6) history of congenital heart defects (repaired or unrepaired). Following the establishment of described preliminary eligibility criteria, patients were invited to participate in the study. Fifty of the contacted patients visited our center and provided their latest echocardiography reports or underwent echocardiography. Patients with isolated diastolic dysfunction, ie, constrictive cardiomyopathy (1 patient with Tb pericarditis) and post-chemotherapy CHF (3 patients), were excluded from the case group. Further 4 patients refused to give blood sample after echocardiography and were excluded. Details on case selection criteria are depicted in Figure 1. All 42 patients were taking stable doses of diuretics, 29 were taking beta-blockers, and 3 were taking digitals.

Patients with ischemic etiology of CHF were defined based on evidence of coronary artery disease identified through catheterization techniques or myocardial perfusion studies (n = 30). The etiology for CHF in the rest of the patients was identified through detailed clinical history taking. Those not fulfilling the criteria for ischemic CHF, and with otherwise non-significant history, were categorized as patients with CHF of non-ischemic origin. Thirty patients had ischemic CHF, while 12 patients had non-ischemic CHF. None of the patients had undergone open heart surgery, resynchronization therapy, or had cardioverter-defibrillators implanted. Thirty patients had both systolic and diastolic heart failure and none of the patients had co-morbid atrial fibrillation. Patients were also classified into four functional classes according to NYHA classification. Six patients belonged to class I, ie, they had no limitation in physical activity, 11 to class II, ie, they had signs of fatigue, palpitation, dyspnea during less-than-ordinary activities, 12 to class III, and 13 to class IV, ie, they experienced dyspnea and fatigue during activities less-than-ordinary living chores or at rest, respectively.

Blood samples for the control group were randomly selected from samples obtained by the Iranian Blood Transfusion Bank during the same period when case collection was performed. Ninety individuals were initially selected from a total of ~ 1500 referrals, and were age- and sex- matched with the cases based on their records. These individuals were contacted by telephone, provided information about the aim of the study, and asked whether they opt for their sample to be used for this purpose. A detailed medical history was then obtained. Controls were excluded if they had any history of coronary artery disease, hypertension, diabetes, or other cardiac risk factors. Disease-free status of individuals with incomplete medical records was confirmed through outpatient visit and echocardiography. This led to inclusion of a final number of 42 age- and sex-matched healthy individuals as control group. Mean systolic/diastolic blood pressure of controls was 135 ± 28 mm Hg /83 ± 21 mm Hg.

This study was approved by local ethics committee and institutional review board of Tehran University of Medical Sciences, under ID: 2034, in July 2015. The study protocol and aim of the study was clarified to all study participants. Signed informed consent forms were obtained from all enrolled case participants, to perform sampling, echocardiography and necessary procedures (at the expense of the investigators) and for publication of the results as papers.

### RNA isolation

An amount of 5 mL of whole blood was obtained from cases and controls and PBMCs were isolated through Ficoll–Paque density gradient centrifugation (Lymphodex, Inno-train, Kronberg, Germany). Total cellular RNA was isolated with 12000 rpm (RPM) centrifugation, using RNX solution (SinaClon, Tehran, Iran), and an amount of 5-10 × 10^6^/mL of the cellular suspension. RNA pellets were melted in nuclease-free DEPS water (30 μL) were stored at -70°C. Quality of extracted RNA was determined using Nanodrop ND-1000 instrument (Thermo Scientific, Waltham, MA, USA), measuring optical density (OD) of the samples at 260 nm and 280 nm. Preparations with an OD ratio 260/280 less than 1.8 were discarded. Phenolic contamination was assessed using OD ratio of 260/230 nm, which was above the optimal (ie, phenolic absorption was low).

### Complementary DNA synthesis

1 µg of total RNA from each sample was reverse-transcribed to complementary DNA (cDNA) using High Capacity cDNA Reverse Transcription Kit (CinnaGen, Tehran, Iran) according to the manufacturer’s instructions. An amount of 500 ng total RNA, 2μL 10X Buffer M-MuLV, 1 μL Random hexamer (100 μM, 0.2μg/μL), 1 μL Oligo d(T)18 primers (100 μM, 0.5μg/μL), 0.5μL M-MuLV Reverse Transcriptase (200u/µL), 0.5 μL RNase inhibitor (40u/µL), 2μL dNTP Mixture, and nuclease-free water were used for each reaction in a total volume of 20 μL. The mixture was incubated at 25°C °for 10 min, followed by 42°C° for 60 min and finally 5 min at 85°C°. cDNA solutions were stored at 80°C until they were used in further analyses.

### Quantitative real-time reverse transcriptase PCR

IL-17 mRNA expression levels were analyzed using quantitative real-time reverse transcriptase PCR (Q-RT-PCR) method. Quantitative PCR reactions were performed with 20-μL reaction mixtures, using TaqMan Universal PCR Master Mix (ABI, London, UK) and 1-μL primer/probe pairs (Applied Biosystems, Foster City, CA, USA). Difference in expression of IL-17 between cases and controls were normalized against β-Actin (as housekeeping gene), via ABI Prism7900 Sequence Detection System (Thermo Fisher Scientific, Mumbai, India). Threshold cycle (CT) during the exponential phase of amplification was determined by real-time monitoring of fluorescent emission after cleavage of sequence-specific probes (Taqman DNA Probes, Applied Biosystems) by nuclease activity of Taq polymerase (Hot start Taq Plus DNA Polymerase 250U, Qiagen, Germany). Results were calculated using the comparative Ct (2^−ΔΔCT^) formula (11).

### Cell preparation and culture conditions

PBMC were resuspended in complete medium (CM) in 10^6^/mL-concentration. This CM consisted of RPMI 1460 medium (Sigma-Aldrich, Taufkirchen, Germany) supplemented with 1% penicillin- streptomycin-L-glutamine (Sigma-Aldrich). Isolated PBMC were incubated at 37°C, with 1.5% phytohemagglutinin (PHA, 15 μg/mL) (Gibco, Gaithersburg, MD, USA). After 48 hours of incubation with PHA and exposure to humidified (95% H2O + 5% CO2) atmosphere, the supernatant was collected and stored at -70°C until enzyme-linked immunosorbent assays (ELISA).

### Measurement of TGF-β1 in culture supernatants

We measured TGF-β1 in the supernatant of PHA-stimulated PBMCs using specific commercial ELISA according to manufacturer’s instructions (eBioscience, Thermo Fisher Scientific, Waltham, MA, USA). Relative concentrations were determined by measuring optical density at 450 nm in a spectrophotometric microtiter plate reader.

### Flow cytometry analysis

To stimulate cytokine production, 2 × 10^6^ cells were incubated in each well of a 24-well plate with phorbol myristate acetate (PMA) (50 ng/mL) (Sigma-Aldrich) and ionomycin (1 μg/mL) (Sigma-Aldrich), at 37°C and CO2 5% for 5 hours. Cultures were excubated at 30 minutes to add Brefeldin A (3.0 μg/mL) (eBioscience, Thermo Fisher Scientific) to inhibit endoplasmic reticulum transportation of cytokines. After 5 hours, cells were extracted and expanded to 50 µL solution at 1 × 10^6^ concentration.

In order to perform cell surface staining, an amount of 1 × 10^6^ cells from each tube of cultured PBMC was suspended in a 5% Fetal Calf Serum (FCS) buffer. Anti-cell-surface antibody, anti-CD4-fluorescein isothiocyanat (FITC) (eBioscience, Thermo Fisher Scientific, product number 317416 and 302606) was added to the staining buffer to reach the volume of 50 µL and further 50 µL of the cellular suspension was prepared. The mixture was incubated in a dark room at 4°C for at least 30 minutes before being washed and resuspended in staining buffer.

Following extracellular staining with CD4-FITC, cellular suspension was mixed in IC Fixation buffer, incubated at dark room at 20°C, mixed with 2 mL of Permeabilization buffer, and centrifuged at 300-400 g at room temperature, with all steps being repeated twice. Finally, fluorescent anti-Human IL-17A PE solution (eBioscience, Thermo Fisher Scientific, 320112) was added to the cellular precipitate and incubated for further 30-60 minutes in dark room at 4°C. The suspension was then mixed with perm buffer before being centrifuged at 300 g. The resulting cellular cocktail was resuspended in staining buffer and was immediately used to obtain flow cytometry data on a Fluorescence Activated Cell Sorter (FACS) Calibur (Pharmacia, Stockholm, Sweden). Other analyses were performed using the free software flowjo 7.6 (LLC, Ashland, OR, USA).

### Statistical analysis

Normality of distribution was determined by Kolmogrov-Smirnov test. Independent *t* test or Mann-Whitney U-test were used for between-group comparisons. Pearson correlation was used to identify correlations between baseline characteristics of patients, including age and disease duration, with variables of interest. Comparisons between NYHA functional class groups were made using one-way ANOVA analysis, with Bonferroni correction for multiple comparisons. The corrected *P* above the cut-off of 0.05 was considered significant. *Post-hoc* analysis was performed using Tukey's honestly significant difference (HSD) when the Leven’s test satisfied the assumption of homogeneity of variance or the Games Howell test when data did not meet the homogeneity criteria.

Multiple regression analysis was used to assess significant predictors of baseline and stimulated expression levels of IL-17 and Th17 count. We considered NYHA functional class (as ordinal parameter), sex and ischemic vs non-ischemic origin of the CHF (as dichotomous parameters), age and disease duration (as interval parameters) in univariate analyses against baseline and stimulated IL-17 expression and Th17 count (8). Variables showing significant association/correlation to the mentioned dependent variables were entered into the regression model. Multivariate normality was investigated by checking normal Q-Q plots of each variable. A stepwise method was devised to enter variables and the probability of F by at least 0.05 was set as inclusion criterion to enter the next variable. Collinearity diagnostics was performed using Durbin-Watson test and homoscedasticity was tested by *post-hoc* plotting of the residua squares against predicted values. *P* < 5% were considered significant for the model as a whole, and for each variable. Statistical analyses were performed using SPSS Statistics for Windows, Version 23.0. (IBM Corp, Armonk, NY, USA, licensed to the Department of Immunology, School of Medicine of Tehran University of Medical Sciences).

## Results

### IL-17A was overexpressed in patients with CHF and among patients with different NYHA stages

**Figure 2:**
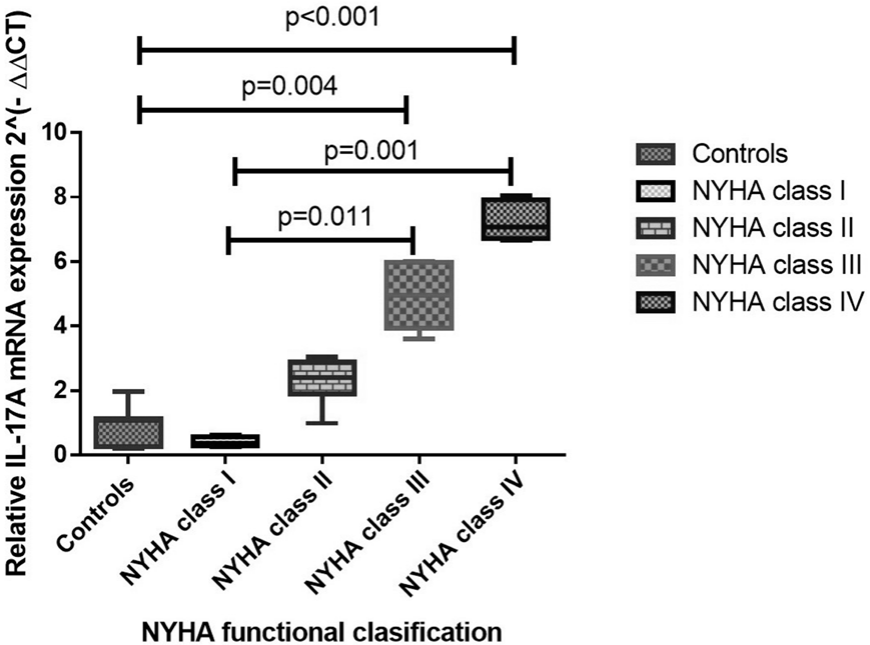
Baseline interleukin 17A (IL-17A) messenger RNA (mRNA) expression in peripheral blood mononuclear cells of patients with chronic heart failure with different functional classes based on New York Heart Association (NYHA) classification. 2^−ΔΔCT^ – comparative delta delta cycle threshold method.

**Figure 3:**
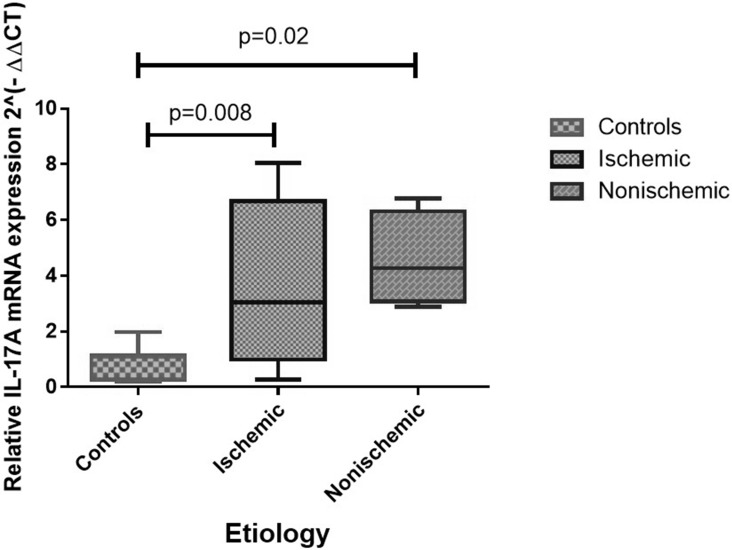
Baseline interleukin 17A (IL-17A) messenger RNA (mRNA) expression in peripheral blood mononuclear cells of patients with chronic heart failure with ischemic vs non-ischemic etiology, 2^−ΔΔCT^ – comparative delta delta cycle threshold method.

IL-17A was higher at baseline level (before stimulation with PHA 1.5%) in patients with CHF compared to controls (*P* < 0.001). One-way ANOVA revealed a significant difference in IL-17A expression among patients with different NYHA groups (F (3, 39) = 35.44, *P* < 0.001). Tukey’s *post-hoc* analysis revealed higher expression in patients with class IV and class III compared to patients with class I (*P* = 0.001 and 0.011, respectively). Patients with classes III and IV combined had higher levels of IL-17A expression than controls (*P* = 0.004 and <0.001, respectively), but not compared with patients with classes I and II (Figure 2). Patients with non-ischemic origin of CHF had higher expression levels of IL-17A than controls. There was no difference in IL-17A expression in patients with ischemic vs non-ischemic origin of CHF (*P* = 0.21) (Figure 3).

### Stimulated IL-17A expression was higher in patients with CHF

**Figure 4:**
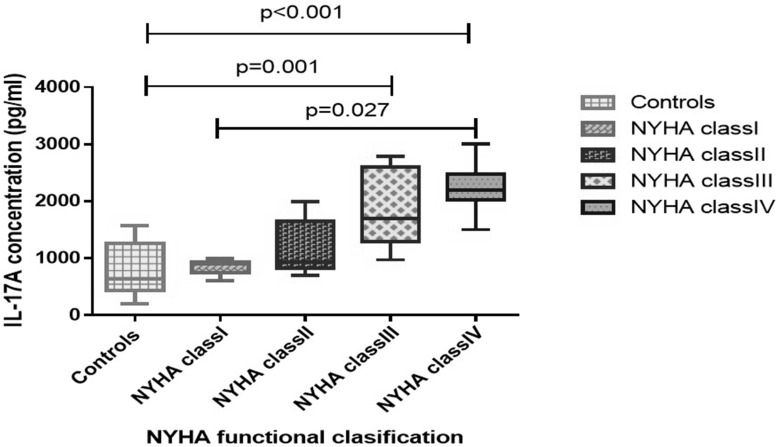
Stimulated interleukin 17A (IL-17A) messenger RNA (mRNA) expression in peripheral blood mononuclear cells of patients with chronic heart failure with different functional classes based on New York Heart Association (NYHA) classification.

IL-17 after stimulation was higher in patients in combined class III and IV compared to controls (Figure 4) (*P* = 0.001 and <0.001, respectively). One-way ANOVA analysis showed that stimulated IL-17A expression was different in patients with different NYHA stages (F (3, 39) = 27.78, *P* < 0.01). Tukey’s *post-hoc* analysis revealed differences in IL-17A expression between NYHA class IV and I (*P* = 0.027) (Figure 4). Both groups, ischemic and non-ischemic CHF, had higher stimulated IL-17 expression than controls (*P* < 0.001 and 0.003, respectively), while there was no difference between the two groups separately (*P* = 0.12)

### T helper 17 population was expanded in patients with CHF and correlated with disease stage

**Figure 5:**
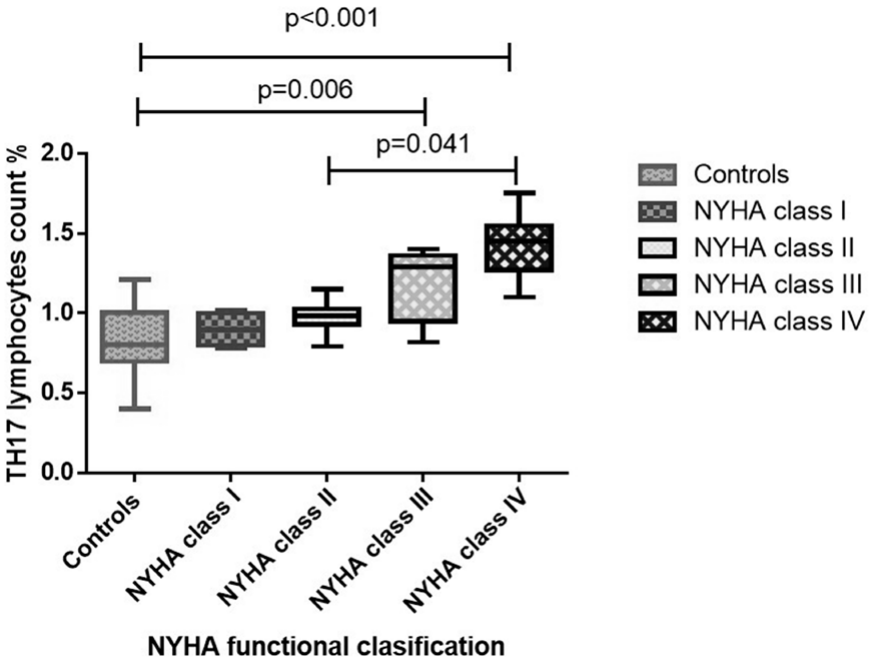
Absolute count of CD4^+^IL-17^+^ cells in patients with chronic heart failure with different functional classes based on New York heart association (NYHA) classification. CD – cluster of differentiation, IL-17 – interleukin 17.

**Figure 6:**
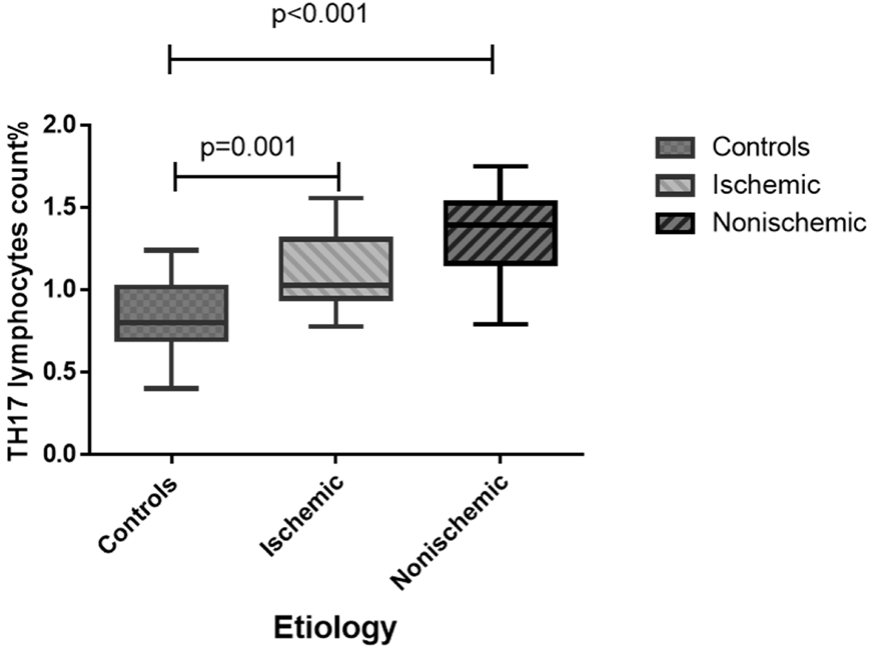
Absolute count of CD4^+^IL-17^+^ cells in patients with chronic heart failure with ischemic vs non-ischemic etiology. CD – cluster of differentiation, IL-17 – interleukin 17.

Th17 cells (CD4^+^IL17^+^) were expanded in patients with CHF (*P* < 0.001). This increase was significantly different between all NYHA classes (F (3, 39) = 14.01, *P* < . 05) and according to post-hoc Tukey between classes IV and II (*P* = 0.041). The difference was also significant between the control group and patients in class III (*P* = 0.006) and IV (*P* < 0.001) (Figure 5). While Th17 count was higher in patients with ischemic origin than in controls (*P* = 0.001) and also in patients with non-ischemic origin than in controls (*P* < 0.001), there was no difference between ischemic and non-ischemic group (*P* = 0.08) (Figure 6).

### Multiple linear regression model

Baseline and stimulated IL-17A expression showed significant correlation with age in both patients (Pearson correlation coefficient: 0.44, *P* < 0.01, and Pearson correlation coefficient: 0.37, *P* = 0.012, respectively) and controls (Pearson correlation coefficient: 0.220, *P* < 0.036, and Pearson correlation coefficient: 0.332, *P* = 0.01, respectively). IL-17A at baseline and after stimulation correlated with disease duration in CHF patients (Pearson correlation coefficient: 0.112, *P* < 0.001, and Pearson correlation coefficient: 0.109, *P* < 0.01, respectively). Male patients with CHF had a borderline lower expression of IL-17 and Th17 count compared to women (*P*: 0.049 and 0.02, respectively).

Finally, we included the significant factors predicting IL-17 expression and Th17 count: age, sex, disease duration, NYHA functional class, and ischemic vs non-ischemic origin of CHF into a multiple regression model. It showed that the NYHA function class was the only meaningful predictor of baseline and stimulated IL-17 and Th17 levels, considering other variables: age, sex, disease duration, and CHF etiology as covariates (*P* = 0.02, <0.001, and 0.033, respectively).

## Discussion

Patients with CHF had higher IL-17A levels both at baseline and post-stimulation, compared to controls. IL-17A levels and the absolute count of Th17 cells were consistently higher in patients with advanced functional stages of CHF (stage III and IV), compared to both controls and patients in functional class I and II. These results suggest that peripheral IL-17 production is associated with CHF progression, and that Th17 cells are perhaps the main source of IL-17 in late stages of CHF. Also, according to our previous results investigating Treg and Th1 population in patients with CHF (12,13), the current study supports the hypothesis that expansion of Th17 cells is a major driving force for progressive cardiac muscle dysfunction in late stage of CHF (14,15). Expansion of the regulatory T-cells during early CHF phases and high TGF-β expression could have immunosuppressive and profibrotic potentials. Meanwhile, expansion of Th17 over Tregs sustains the initial proinflammatory milieu due to Th1 cytokines and prevents regulatory T-cells from exhibiting anti-inflammatory features (16).

As CHF progresses, the Th17/Treg ratio progressively increases to support the profibrotic state, with activation of p38/mitogen-activated protein kinases (1). This supports the finding of the current study and the role of IL-17 as the major driving force for cardiac remodelling in later stages of CHF (5). Yan et al (17) reported almost the same findings in a mouse model of acute myocardial infarction, where they demonstrated increased survival after day 7 in mice lacking either interleukin 23 (IL-23), IL-17A, or T-cell receptor gamma delta (TCRγδ) positive T-cells. TCRγδ is known in the literature to mediate an antigen-specific slowly-evolving immune response during later stages of T-cell maturation and have a terminator effect on the immune response. Interestingly, the expanded TCRγδ T-cells produce IL-17 in response to IL-23 and IL-1β after the acute inflammatory phase post infarction, along with increased population of the proinflammatory M1 macrophages on the periphery. M1 macrophages are perhaps responsible for IL-23 production, while further mediating the infiltration of IL-17A-producing TCRγδ T-cells into the myocardium, which promotes myocardiocyte apoptosis and fibroblast proliferation. IL-17 and activated fibroblasts facilitated accentuated infiltration of cytokine-producing monocytes/macrophages into the lesion site (17), providing a positive feedback loop. IL-17 is even able to stimulate cardiac fibroblasts to secrete chemotactic agents for monocytes/macrophages, facilitating their migration into the myocardium and their maturation via granulocyte-macrophage colony-stimulating factor (18). Blocking the IL-23/IL-17A axis has been shown to prevent dilated cardiomyopathy and late-onset ventricular remodelling in both cases (19,20)

Restoring the balance between Th17/Treg should be able to reverse the positive feedback loop described above. Even in rats with established ischemic cardiomyopathy, physical training was able to restore the Treg population and mitigate the production of IL-6, TNF-α, and later IL-17 in peripheral blood (21). Catechin, a cardioprotective agent, and 25-hydroxy vitamin D, have been reported to exert the same effect by regulating the balance between IL-17/IL-10 production in mouse models of ischemic cardiomyopathy (13,22).

Finally, we found no difference between patients with ischemic and those with non-ischemic origin of CHF in IL-17 levels or Th17 cell count. It might be postulated that the late increase in IL-17 happens regardless of the initial insult and as a product of Th17/Treg induction dysregulation. In line with this, IL-17 was able to drive myeloid cell infiltration and remodelling in models of inflammation-induced dilated cardiomyopathy (18), and even in an experimental model of autoimmune myocarditis (15). Our results were, however, confined to a group with predominantly ischemic origin (30 out of 42) of CHF, and cannot provide reliable evidence on alteration of IL-17 expression and Th17 count in patients with non-ischemic CHF. The calculated *post-hoc* power of the comparisons between patients with ischemic and non-ischemic CHF was 84.01%, 89.0%, and 89.5% for baseline IL-17A, stimulated IL-17A, and Th17, respectively. Meanwhile, the low number of patients with non-ischemic CHF does not affect the assumption that with a larger group of CHF patients, IL-17 could be found to be preferentially expressed in either of the two groups.

Results of our study are limited in validity by two main sources: 1) selection of the case group (selection bias) and 2) measurement bias. Our effort was focused on selecting patients with CHF in whom the inflammatory/immune status was not disrupted by any other accompanying condition. Meanwhile, we could not control for the anti/pro-inflammatory effects of diuretics, diabetes medication, and digitals in general inflammatory milieu of the body, cytokine production, and T-cell population. Also, we did not perform perfusion scan in all patients, which is considered a gold standard to discriminate left-sided from right-sided heart failure. Echocardiography is generally acceptable to measure volume and pressure of heart chamber and differentiate systolic from diastolic dysfunction. As for measurement bias, we were unable to differentiate between local (within cardiac muscle) and peripheral source of IL-17 production. We know that in late stages of CHF, infiltrating lymphocytes are also a major source of cytokine production and contribute to cardiac dysfunction and failure. We were unable to differentiate between these two sources.

Nonetheless, our results reiterate the importance of IL-17A as a principal dual-effect, profibrotic and proinflammatory, cytokine in late stages of CHF. It would be also useful to use other cell surface markers of T-cells to identify cell-specific targets for development of biologic agents to combat cardiac remodeling in advanced CHF.
